# The Balance of MFN2 and OPA1 in Mitochondrial Dynamics, Cellular Homeostasis, and Disease

**DOI:** 10.3390/biom15030433

**Published:** 2025-03-18

**Authors:** Paola Zanfardino, Alessandro Amati, Mirko Perrone, Vittoria Petruzzella

**Affiliations:** Department of Translational Biomedicine and Neurosciences (DiBraiN), University of Bari Aldo Moro, Piazza Giulio Cesare, 70124 Bari, Italy; paola.zanfardino@uniba.it (P.Z.); a.amati17@alumni.uniba.it (A.A.); m.perrone97@studenti.uniba.it (M.P.)

**Keywords:** mitochondria, mitochondrial dynamics, neurodegenerative diseases, autophagy, mitophagy, oxidative phosphorylation, proliferation, senescence, mTOR signaling

## Abstract

Mitochondrial dynamics, governed by fusion and fission, are crucial for maintaining cellular homeostasis, energy production, and stress adaptation. MFN2 and OPA1, key regulators of mitochondrial fusion, play essential roles beyond their structural functions, influencing bioenergetics, intracellular signaling, and quality control mechanisms such as mitophagy. Disruptions in these processes, often caused by MFN2 or OPA1 mutations, are linked to neurodegenerative diseases like Charcot-Marie-Tooth disease type 2A (CMT2A) and autosomal dominant optic atrophy (ADOA). This review explores the molecular mechanisms underlying mitochondrial fusion, the impact of MFN2 and OPA1 dysfunction on oxidative phosphorylation and autophagy, and their role in disease progression. Additionally, we discuss the divergent cellular responses to MFN2 and OPA1 mutations, particularly in terms of proliferation, senescence, and metabolic signaling. Finally, we highlight emerging therapeutic strategies to restore mitochondrial integrity, including mTOR modulation and autophagy-targeted approaches, with potential implications for neurodegenerative disorders.

## 1. Introduction

Mitochondria are dynamic organelles constantly undergoing fusion and fission to maintain cellular balance and adapt to metabolic needs. These processes, known as mitochondrial dynamics, are essential for preserving the mitochondrial structure, optimizing energy production, and supporting intracellular signaling. By responding to environmental and physiological signals, mitochondria adjust their shape to help cells cope with stress and energy fluctuations [1,2]. Fusion is mediated by GTPase proteins, including mitofusins (MFN1 and MFN2) in the outer mitochondrial membrane (OMM) and optic atrophy 1 (OPA1) in the inner mitochondrial membrane (IMM) [3,4]. These proteins maintain mitochondrial elongation, preserve membrane potential, and regulate oxidative phosphorylation (OxPhos) and cellular quality control mechanisms like mitophagy. These processes are particularly crucial in high-energy tissues such as the brain, where neuronal health and function heavily depend on mitochondrial dynamics [5,6]. Conversely, fission is primarily driven by dynamin-related protein 1 (Drp1) [7,8]. Beyond their well-characterized roles in mitochondrial fusion, MFN2 and OPA1 influence broader cellular processes [9,10,11]. Disruptions in mitochondrial dynamics, often caused by mutations in MFN2 (OMIM #608507) or OPA1 (OMIM #605290), are associated with several neurodegenerative diseases and hereditary neuropathies, such as Charcot-Marie-Tooth disease type 2A (CMT2A, OMIM #609260) and autosomal dominant optic atrophy (ADOA, OMIM #165500). This review explores the structural and functional complexities of MFN2 and OPA1, focusing on their roles in mitochondrial quality control and bioenergetics and their involvement in cellular differentiation and metabolic signaling. By highlighting emerging therapeutic strategies targeting these proteins, we aim to enlighten their potential to restore mitochondrial integrity and cellular homeostasis in the context of neurodegenerative diseases.

## 2. Structural and Functional Insights into MFN2 and OPA1

MFN2 and OPA1 possess sophisticated structures and undergo precise regulation, enabling their diverse functions.

### 2.1. MFN2: Structure and Function

MFN2 is a nuclear-encoded protein localized to the outer mitochondrial membrane (OMM) [12]. The *MFN2* gene (OMIM #603560) resides on chromosome 1 at position 36.22, encompassing 19 exons and producing two transcript variants. These variants encode two protein isoforms: Isoform 1 (757aa; 86,402 Da) and Isoform 2 (436aa; 50,041 Da). MFN2 contains several conserved functional domains arranged sequentially from the N-terminal to the C-terminal: a GTPase domain, two coiled-coil domains (HR1 and HR2), a proline-rich domain, and two transmembrane domains [13]. The GTPase domain is critical in mediating the fusion process, with motifs G1 to G5 forming the catalytic center and coordinating GTP binding and hydrolysis [14,15]. The HR2 domain enables tethering of adjacent mitochondria through an antiparallel coiled-coil dimeric structure, forming both homotypic (MFN1–MFN1 or MFN2–MFN2) and heterotypic dimers (MFN1–MFN2) [16,17]. Additionally, MFN2 possesses a unique N-terminal P21 Ras-binding domain, distinguishing it functionally from its homolog MFN1 [18]. While MFN1 is predominantly expressed in the heart and testis, MFN2 is abundant in the brain, underscoring its specialized role in neural tissue [19]. Crystal structures of MFNs in both monomeric and dimeric states reveal a dynamin-related GTPase domain and an adjacent four-helical bundle. This bundle comprises helices α1 and α2 from the N-terminal region, helix α3 following the G-domain, and helix α4 from the C-terminal region, connected to α3 via a linker [20,21,22]. Structural studies indicate that MFNs undergo dynamic conformational changes, transitioning between resting and tethering-permissive states. In the resting state, MFNs remain in a closed conformation, preventing tethering due to antiparallel HR1–HR2 interactions and the tight association of the GTPase domain with the OMM. In the tethering-permissive state, these intramolecular interactions weaken, allowing the HR2 domain to extend into the cytosol, where it can engage with HR2 domains of MFNs on opposing membranes. This structural transition facilitates mitochondrial tethering and fusion, as previously proposed [23].

### 2.2. OPA1: Isoforms and Functional Domains

OPA1, a nuclear-encoded protein located in the inner mitochondrial membrane (IMM), is encoded by the OPA1 gene (OMIM #605290) on chromosome 3q29. This gene spans over 60 kb and produces eight isoforms through alternative splicing, primarily involving exons 4, 4b, and 5b [24]. Key isoforms include Isoform 1: 960aa (111,631 Da), Isoform 2: 997aa (115,884 Da), and Isoform 3: 961aa (111,822 Da). OPA1 contains several functional regions, including an N-terminal mitochondrial targeting sequence (MTS), a GTPase domain, two coiled-coil domains, and a GTPase effector domain (GED). These regions are critical for anchoring OPA1 to the IMM, mediating protein–protein interactions, and enabling GTP-dependent fusion activity [25]. Beyond the GTPase, the bundle signaling element (BSE) and stalk domains are additional important protein parts. The GTPase domain has a central β-sheet surrounded by α-helices, while the BSE is made of three helices, with hydrophobic residues in the core and charged residues on the surface. The stalk domain of OPA1 consists of four α-helices arranged in an extended bundle, helping OPA1 interact with and remodel membranes. Studies of OPA1’s tertiary structure revealed that OPA1 forms helical filaments, with tightly packed stalk domains providing stability, while the GTPase domains remain flexible, allowing for structural changes. Three-dimensional reconstructions showed these filaments vary in diameter and can adopt different forms, more or less compacted. These findings suggest that OPA1 filaments are flexible and can change shape, especially when interacting with cardiolipin-rich membranes. This flexibility is crucial in OPA1’s role in mitochondrial fusion and membrane remodeling [17].

After mitochondrial import, OPA1 undergoes proteolytic cleavage at specific sites. Under stress conditions, OMA1 cleaves at site S1 (corresponding to exon 5). YME1L cleaves at site S2 (corresponding to exon 5b) during normal conditions [26]. This processing generates two OPA1 forms: long OPA1 (l-OPA1) and short OPA1 (s-OPA1). While l-OPA1 anchors to the IMM, s-OPA1 resides in the intermembrane space (IMS). Both forms are essential for mitochondrial fusion, with l-OPA1 facilitating homotypic interactions and s-OPA1 enhancing the fusion process [27].

### 2.3. MFN2 and OPA1 in the Mitochondrial Fusion Process

Mitochondrial fusion involves several key proteins and interactions across the outer and inner mitochondrial membranes (OMM and IMM) [6,28]. The fusion of the OMM is initiated by the mitofusins MFN1 and MFN2, which tether two mitochondria through interactions between their HR2 domains. This tethering is followed by GTP hydrolysis-induced conformational changes, driving membrane fusion. While MFN1 is primarily responsible for completing OMM fusion, MFN2 plays a supportive role, including mediating OMM fusion and regulating mitochondria–endoplasmic reticulum (mito-ER) contact sites, also known as the ER mitochondria-associated membranes (MAMs). Several processes known to be regulated or directly occurring at MAM have been reported to be influenced by the presence of MFN2 (see paragraphs 4 and 6). Recent structural studies of MFN1 and MFN2 have provided mechanistic insights into their roles in mitochondrial fusion [22,29]. However, the physiological relevance remains under investigation due to the absence of the transmembrane domain in these structures and uncertainties surrounding their topology [30]. Once OMM fusion is complete, IMM undergoes fusion, a process mediated by OPA1. l-OPA1 facilitates homotypic interactions between membranes but requires s-OPA1 to complete the fusion process. Studies have demonstrated that l-OPA1 interacts with cardiolipin, a mitochondrial-specific phospholipid, to form trans complexes capable of driving membrane fusion. s-OPA1 enhances this process, indicating that both isoforms are essential for IMM fusion [27,31]. The coordination of OMM and IMM fusion remains an unresolved aspect of mitochondrial dynamics. One model suggests that MFN1-mediated GTP hydrolysis stimulates the oligomerization of OMM fusion pores, subsequently facilitating IMM fusion. OPA1 may then form right-turned helical assemblies, potentially activated by MFN1, to drive IMM fusion [32]. This coordinated interaction ensures that mitochondrial fusion occurs seamlessly, maintaining organelle integrity and function. Mitochondrial fusion is crucial in maintaining mitochondrial quality by selectively preventing the reintegration of defective mitochondria with lower membrane potential [33]. Additionally, it facilitates the exchange of mitochondrial DNA (mtDNA) and proteins, diluting damage and supporting mitochondrial health [34]. In the presence of mtDNA mutations, fusion helps mitigate defects in respiratory chain proteins, highlighting its importance in sustaining bioenergetic efficiency [35]. Disruption of this process due to the loss of key fusion regulators, such as MFN1, MFN2, or OPA1, leads to a decrease in mtDNA copy numbers and an increase in mitochondrial dysfunction [36].

## 3. Mitochondrial Fusion Proteins in the Regulation of Oxidative Phosphorylation

The efficiency of OxPhos—the process of producing ATP via the electron transport chain (ETC)—is tightly regulated by mitochondrial dynamics. MFN2 and OPA1, key proteins governing mitochondrial fusion and *cristae* structure—invaginations of the inner mitochondrial membrane where respiratory chain complexes are located—directly influence bioenergetic efficiency and cellular metabolic states. Elongated mitochondria, formed through fusion mediated by MFN2 and OPA1, enhance OxPhos efficiency and ATP production [37,38]. In contrast, fragmented mitochondria often exhibit impaired OxPhos, reduced mitochondrial DNA (mtDNA) content, and elevated reactive oxygen species (ROS) levels, contributing to cellular dysfunction [39,40,41]. Recent research highlights the adaptability of mitochondria under metabolic stress. During nutrient deficiency, mitochondria dynamically segregate into specialized subpopulations: ATP-producing units and biosynthetic hubs. One subpopulation, devoid of *cristae* but enriched in pyrroline-5-carboxylate synthase (P5CS), supports proline and ornithine biosynthesis, optimizing metabolic flexibility through fusion–fission cycles [42]. This segregation exemplifies how mitochondrial dynamics align with cellular metabolic demands.

### 3.1. MFN2: A Key Regulator of Fusion, Bioenergetics and Beyond

**Figure 1:**
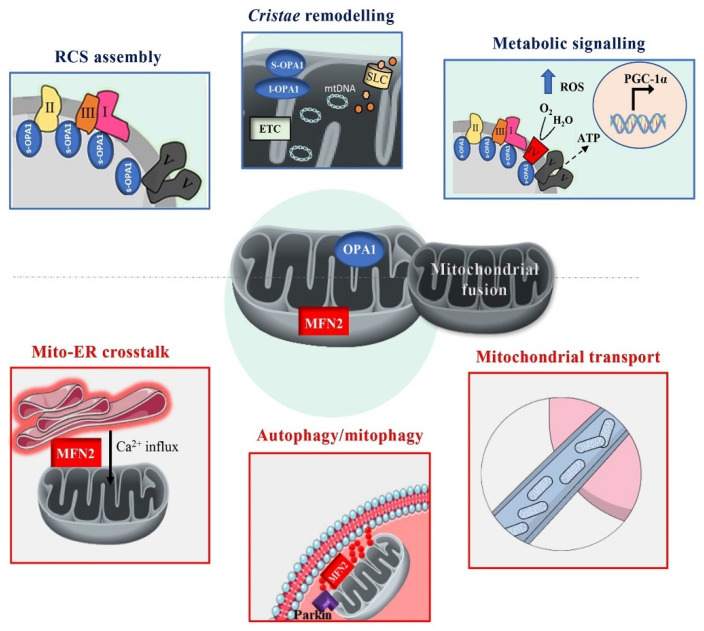
Roles of MFN2 and OPA1 proteins in cellular bioenergetics. This figure illustrates the key mechanisms and functions of MFN2 and OPA1 in cellular bioenergetics. MFN2 (red) plays a pivotal role in outer mitochondrial membrane (OMM) fusion and mediates mitochondria-ER crosstalk, facilitating calcium transfer to mitochondria. This calcium influx modulates respiratory chain complex (RCC) activity, optimizing ATP production. Additionally, MFN2 regulates autophagy and mitophagy, ensuring mitochondrial quality control by selectively removing dysfunctional mitochondria. PINK1-phosphorylated MFN2 acts as a Parkin receptor, tagging damaged mitochondria for degradation. By maintaining a healthy mitochondrial network, MFN2 supports bioenergetic efficiency and prevents metabolic stress. Moreover, MFN2 contributes to mitochondrial transport along axons, ensuring that neurons meet their high-energy demands. OPA1 (blue), in contrast, is crucial for inner mitochondrial membrane (IMM) fusion and the assembly of respiratory chain supercomplexes (RCS), which enhance ATP production while minimizing ROS generation. It also regulates *cristae* morphology, a key determinant of oxidative phosphorylation (OxPhos) efficiency and interacts with mitochondrial solute carriers (i.e., SLC25A), sensing substrate availability and adjusting mitochondrial function. OPA1 dysfunction disrupts these processes, leading to metabolic signaling alterations, including increased ROS levels, which can activate nuclear responses via PGC-1α. MFN2 and OPA1 coordinate mitochondrial fusion, morphology, and bioenergetic regulation, ensuring proper cellular function and adaptation to metabolic demands.

MFN2 is a critical regulator of mitochondrial fusion and bioenergetics, and its role extends beyond mere mitochondrial dynamics (Figure 1). Alterations in mitochondrial fusion, often caused by impaired MFN2-GTPase activity, lead to severe metabolic dysfunctions, including suppressed OxPhos, diminished ATP production, and mtDNA instability. Such dysfunctions are implicated in pathological conditions such as neurodegeneration and metabolic syndromes [40]. As a tethering factor, MFN2 facilitates direct communication between the mitochondria and the ER [43,44]. This interaction is crucial in maintaining cellular bioenergetics by limiting ROS production, alleviating ER stress, and facilitating calcium transfer from the ER to mitochondria. These processes sustain NADH levels, which are critical for OxPhos [45,46]. Furthermore, MFN2-mediated fusion enhances OxPhos during periods of high metabolic demand, such as cell proliferation, though proliferative cells often shift to glycolysis to meet biosynthetic needs [37,47]. Despite the central role of MFN2 in tethering, conflicting findings exist regarding the effects of MFN2 loss on mito-ER contacts. Some studies suggest that the loss of MFN2 reduces these contacts, impairing mitochondrial function and increasing cellular stress [48]. In contrast, other studies suggest that the absence of MFN2 may increase these contacts, complicating the interpretation of its precise role in metabolic regulation [49]. A detailed discussion of this controversy is beyond the scope of this review and can be found in other literature [50,51]. In addition to its functions in mitochondrial dynamics, MFN2 is involved in mitochondrial transport along axons, a process essential for ensuring neurons meet their energetic and metabolic demands [52]. MFN2 facilitates the attachment of mitochondria to microtubules via interactions with motor proteins like Miro and Milton, ensuring efficient mitochondrial distribution [53]. Interestingly, while MFN2 knockout impairs mitochondrial transport along axons, similar defects are not observed in OPA1-deficient neurons, despite both conditions causing comparable mitochondrial fragmentation [10,54]. This distinction highlights MFN2’s multifaceted role, extending beyond mitochondrial fusion to influence a broader spectrum of cellular processes that support cellular metabolism and energy production [9].

### 3.2. OPA1: Master Regulator of Cristae Structure and Supercomplexes Assembly

OPA1 is central in maintaining mitochondrial *cristae* structure, organizing respiratory chain complexes (RCCs) into supercomplexes (RCS) (Figure 1). These supercomplexes optimize electron transfer and maximize OxPhos efficiency, highlighting the importance of *cristae* integrity in bioenergetics [55,56]. Overexpression of OPA1 in mice enhances *cristae* integrity, improving OxPhos and ATP production, while its knockdown disrupts mitochondrial membrane potential and respiration, underscoring its essential role in mitochondrial function [56]. OPA1 acts as a metabolic sensor, interacting with solute transporters in the inner mitochondrial membrane (IMM) to regulate *cristae* remodeling in response to cellular energy demands. This remodeling is independent of OPA1’s fusion activity and is crucial for maintaining respiration and cell survival during metabolic stress, such as nutrient deprivation [56]. Studies suggest OPA1 interacts with mitochondrial solute carriers (i.e., SLC25A), sensing substrate availability and adjusting mitochondrial function accordingly, thus bridging bioenergetics and metabolic signaling [57]. Modulating these proteins could enhance OxPhos efficiency, reduce ROS production, and improve cellular resilience under metabolic stress. Additionally, understanding the role of mitochondrial solute carriers in OPA1-mediated *cristae* remodeling offers new avenues for designing interventions tailored to specific metabolic conditions.

### 3.3. Metabolic Cues Driving Mitochondrial Fusion

Mitochondrial bioenergetics is essential for cellular function, linking metabolic signals to regulate mitochondrial fusion and fission [28,58]. This dynamic nature allows mitochondria to adapt to fluctuating cellular demands by integrating their activity with physiological processes and shaping metabolism. Under nutrient scarcity, mitochondria undergo significant remodeling, characterized by reduced *cristae* density, respiratory capacity, and size, alongside increased contact with the ER. These changes facilitate calcium and lipid flux, enabling the cell to balance energy under metabolic stress [38,59]. Proteolytic inactivation of OPA1, mediated by MFN2, further promotes mito-ER contact formation, underscoring the importance of mitochondrial dynamics in stress adaptation [59,60].

### 3.4. Regulation by Metabolic Sensor Kinases and Post-Translational Modifications

Metabolic sensor kinases, including mTOR, AMPK, and PKA, dynamically regulate mitochondrial fusion and fission by integrating extracellular signals such as nutrients, oxygen, and growth factors [61,62]. For example, mTOR signaling enhances mitochondrial fusion by modulating OPA1 expression and activity, while AMPK activation during nutrient deprivation suppresses mTORC1 and promotes fusion, increasing energy efficiency [38,62,63,64]. Post-translational modifications (PTMs), such as phosphorylation, ubiquitylation, and sumoylation, provide an additional layer of regulation. Phosphorylation of Drp1 at Ser637 by PKA inhibits its fission activity, promoting mitochondrial hyperfusion [65]. Meanwhile, ubiquitylation of MFN2 by E3 ligases modulates its fusion activity in response to specific stimuli, enabling precise adaptation of mitochondrial dynamics to cellular needs [66].

## 4. Dynamic Changes in Mitochondrial Structure and Quality Control

Dynamic changes in mitochondrial structure are central to quality control mechanisms that maintain cellular and mitochondrial health [67]. These mechanisms enable the mitochondrial network to adapt to challenges such as protein misfolding, oxidative stress, and organelle damage [68,69]. Mitochondrial quality control encompasses transcriptional responses mediated by factors that shuttle between mitochondria and the nucleus—such as those involved in retrograde signaling and the mitochondrial unfolded protein response (mtUPR)—as well as stress-induced mitochondrial hyperfusion (SIMH), which enhances mitochondrial resilience under acute stress. These mechanisms also involve the degradation of misfolded proteins, regulating mitochondrial proteostasis, and the selective removal of damaged mitochondria through processes like mitophagy and autophagy [70,71].

### 4.1. Mitochondrial Retrograde Signaling

Mitochondria contain multiple copies of a small genome, the mitochondrial DNA (mtDNA), with most of the mitochondrial proteome encoded by nuclear genes regulated by transcription factors (TFs) and their associated cofactors [72,73,74,75]. Thus, maintaining mitochondrial homeostasis critically depends on nuclear transcription and signaling pathways that inform the nucleus of mitochondrial dysfunctions and changes in cellular metabolism [76,77]. This regulation occurs partly through nuclear TFs and cofactors modulated by cytosolic mediators such as ROS and the NAD/NADH ratio [78,79]. When mitochondrial dysfunction occurs, retrograde signaling pathways convey mitochondrial distress to the nucleus, triggering adaptive changes in nuclear gene expression and cellular physiology [80,81].

### 4.2. Stress-Induced Mitochondrial Hyperfusion (SIMH)

Acute stressors, such as oxidative damage or starvation, trigger SIMH, a protective response characterized by elongated mitochondrial networks. SIMH enhances bioenergetic efficiency, ATP production, and cellular resilience under stress conditions [82,83]. This response is regulated by the inhibition of Drp1, suppressing mitochondrial fission, and the activation of OPA1 and MFN1, promoting fusion [33,83]. Notably, SIMH prevents mitophagy during nutrient deprivation, preserving mitochondrial function and sustaining energy production [33,84]. Additionally, it enhances calcium buffering, reduces ROS, and supports oxidative metabolism [82]. In contrast, nutrient excess induces mitochondrial fragmentation, shifting metabolism from oxidative phosphorylation to glycolysis, leading to lipid accumulation and metabolic dysfunction [1]. Fragmented mitochondria produce excessive ROS, contributing to oxidative stress and cellular damage [85,86]. One proposed mechanism is that excessive fragmentation disrupts IMM and *cristae* structure, impairing ETC complexes and hindering electron transfer and proton transport [87,88,89]. This electron and proton leakage decreases ATP production while increasing ROS levels and activates Drp1, further promoting mitochondrial fission and perpetuating oxidative stress in a positive feedback loop [90,91].

### 4.3. Integrated Stress Response (ISR) as a Key Regulator of Stress Adaptation

Mitochondria play a pivotal role in the integrated stress response (ISR), a conserved mechanism that adjusts metabolism and protein synthesis under stress conditions. Triggered by mitochondrial dysfunction, nutrient deprivation, or oxidative stress, the ISR downregulates general protein synthesis while selectively upregulating stress-adaptive genes to conserve energy and enhance cellular resilience [92,93]. For instance, OPA1 deficiency in muscle cells activates the ISR, leading to mitochondrial fragmentation, suppression of mTORC1 signaling, and stimulation of AMP-activated protein kinase (AMPK) activity [84]. This dual regulation reduces anabolic processes like protein synthesis while promoting catabolic pathways such as fatty acid oxidation to restore energy balance [94]. Moreover, genetic inhibition of mitochondrial function or treatment with mitochondria-targeted antioxidants can activate AMPK signaling, triggering mitochondrial fragmentation and inducing catabolic pathways as a survival mechanism [95]. In response to mitochondrial injury, AMPK activation can also increase the expression of fusion proteins such as MFN1 and OPA1, promoting a hyper-tubular mitochondrial state that enhances OxPhos and ATP production under stress conditions [35,96,97]. The role of AMPK in mitochondrial dynamics varies depending on the cellular context, the type of stress, the metabolic state, and specific cellular demands. Notably, chronic ISR activation and impaired mitochondrial fusion are linked to neurodegenerative diseases such as Alzheimer’s and Parkinson’s [98,99,100,101,102]. These mechanisms highlight the intricate relationship between mitochondrial dynamics and stress adaptation, underscoring their critical role in maintaining cellular function and homeostasis.

The mitochondrial unfolded protein response (mtUPR) exemplifies a crucial pathway for maintaining mitochondrial proteostasis [103,104]. Protein homeostasis depends on a balance between unfolded proteins and the folding capacity of cellular compartments. Mitochondria possess their chaperones and proteases for quality control; however, when misfolded proteins exceed this capacity, mitochondrial dysfunction ensues, triggering mtUPR to enhance protein folding and degradation [105,106]. This retrograde signaling activates the transcription of nuclear-encoded mitochondrial chaperones and proteases, sustaining mitochondrial proteostasis. While not strictly a quality control mechanism, retrograde communication mitigates mitochondrial dysfunction, preventing further cellular damage [104,107].

### 4.4. Proteolytic Systems and Mitochondrial Proteostasis

The ubiquitin–proteasome system (UPS) plays a crucial role in maintaining mitochondrial proteostasis by clearing defective proteins and facilitating the replacement of non-functional components [104,108]. In addition to its degradative function, the UPS supports protein transport across the mitochondrial outer membrane by targeting import-deficient polypeptides for ubiquitination, marking them for proteasomal degradation [104,109]. Recently, it has been observed that disruption of mitochondrial protein import triggers a quality control mechanism in which YME1L1, the ATP-dependent protease involved in OPA1 cleavage, degrades unassembled translocase components, preventing precursor stalling in the translocase of the outer membrane complex. This process is a protective response to maintain mitochondrial quality control and cellular viability. Import plugging leads to a cell growth defect, and the loss of YME1L1 in yeast exacerbates this inhibition, underscoring the protective role of YME1L1 [110].

## 5. Autophagy/Mitophagy and Mitochondrial Dynamics

Beyond the coordinated action of chaperones and proteases, mitochondrial quality control relies on autophagy, a fundamental mechanism for degrading dysfunctional organelles and protein aggregates. Autophagy can be non-selective, ensuring survival during nutrient deprivation, or selective, targeting specific cellular components such as mitochondria—a process known as mitophagy [111,112,113,114]. This process begins with phagophore formation, likely originating from the ER, trans-Golgi, or endosomes, which expands to engulf damaged components, forming an autophagosome that subsequently fuses with a lysosome to create an autolysosome, where cargo degradation occurs through hydrolases [115,116]. Mitophagy plays a crucial role in mitochondrial turnover and proteostasis, preventing the accumulation of dysfunctional organelles that could otherwise compromise cellular homeostasis [117,118]. A key regulator of this pathway is the PINK1/Parkin system, where PINK1 accumulates on the OMM of damaged mitochondria, recruiting Parkin, an E3 ubiquitin ligase, which ubiquitinates MFNs, leading to mitochondrial fragmentation and clearance [119,120,121,122,123,124,125]. Unfolded mitochondrial proteins can trigger mtUPR and activate PINK1/Parkin-dependent mitophagy, reinforcing mitochondrial quality control [111]. Additionally, PINK1-phosphorylated MFN2 serves as a Parkin receptor, marking mitochondria for degradation [10], while Parkin-mediated MFN2 inhibition regulates ER-mitochondria cross-talk, influencing autophagic signaling [122,126]. This integrated network of mtUPR, UPS, autophagy, and mitophagy ensures that mitochondrial function is preserved, preventing cellular damage and dynamically adapting mitochondrial activity to metabolic demands under physiological and stress conditions [117,118]. Mitochondrial dynamics dictate whether mitochondria undergo fusion, preserving their function, or fragmentation, facilitating mitophagy [127]. During mitophagy, fragmentation exposes “*eat me*” signals, promoting autophagosome formation and lysosomal degradation [128]. In contrast, fusion prevents degradation during periods of nutrient scarcity, forming interconnected mitochondrial networks that enhance ATP production [129]. OPA1 is a crucial regulator of this balance, interacting with FUNDC1 and BNIP3 to mediate autophagy and mitophagy [130,131]. Moreover, mitochondrial-derived vesicles (MDVs) selectively remove damaged mitochondrial components, predominantly occurring at MAMs, where MFN2 localizes and orchestrates mitochondrial fusion and autophagosome formation [115,132,133]. MFN2 depletion disrupts MAM integrity, impairing starvation-induced autophagy and hindering autophagosome formation [133,134]. Furthermore, MFN2 deletion in cardiomyocytes and mouse embryonic fibroblasts leads to autophagosome accumulation, as it blocks autophagosome–lysosome fusion, a crucial step in autophagic degradation, ultimately resulting in metabolic dysfunction, defective lipid metabolism, and reduced mitochondrial oxidative phosphorylation [135,136,137]. These findings highlight the critical role of MFN2-Rab7 interactions, which regulate autophagosome–lysosome fusion, further linking mitochondrial dynamics to autophagic regulation [135]. More recently, MFN2 has been identified as an interactor with novel autophagy-related partners, including RAB5C, a key endosomal regulator of mitochondrial homeostasis, and SLC27A2 [138].

## 6. Mitochondrial Dynamics in Neuronal Differentiation and Bioenergetics

Mitochondrial dynamics are essential for neuronal differentiation, given the high energy demands of neurons. These processes support critical functions such as synaptic transmission, axonal transport, signal propagation, calcium flux, and glutamate cycling [134]. Unlike astrocytes, which predominantly rely on glycolysis, neurons depend on oxidative phosphorylation, highlighting the metabolic diversity within the nervous system [135,136]. Proteomic analyses further confirm this distinction, showing that neuronal mitochondria possess specialized respiratory chain complexes adapted for oxidative metabolism, whereas astrocytic mitochondria exhibit a reduced OxPhos capacity. During neuronal differentiation, mitochondria undergo significant remodeling, which includes alterations in their abundance, localization, and metabolic activity. This process is tightly regulated by MFN2 and OPA1, which facilitate the metabolic transition from glycolysis to oxidative phosphorylation (OxPhos), a crucial step for differentiation [139,140,141,142,143,144]. MFN2 plays a particularly critical role by optimizing bioenergetics, maintaining mitochondrial integrity, and regulating key signaling pathways, such as PI3K-Akt. Silencing *MFN2* disrupts mitochondrial function and differentiation, while its overexpression enhances mitochondrial efficiency and promotes differentiation in neural progenitor cells [145,146]. Interestingly, silencing *MFN2* paradoxically enhances neural differentiation by increasing Akt phosphorylation in embryonic stem cells, suggesting a context-dependent regulatory role [145]. This context dependency extends beyond neural progenitors to other cell types, such as during embryonic stem cell cardiac differentiation, where elevated MFN2 and reduced OPA1 levels promote mitochondrial elongation, enhancing OxPhos to meet the increased energy demands of mature cells [147]. The same oxidative shift also occurs in muscle precursor differentiation [148], human embryonic stem cell mesendoderm commitment [149], spontaneous differentiation of human embryonic stem cells [150], and during spermatogonial differentiation [151]. Specifically, in mesenchymal stem cell (MSC) differentiation, mitochondrial elongation (through increased Mfn1 and Mfn2 expression) is associated with adipogenesis and osteogenesis while mitochondrial fragmentation plays a role in chondrogenesis, partly due to the functional involvement of Drp1 in chondrogenic commitment [149]. This highlights the direct influence of mitochondrial morphology on cellular bioenergetics during differentiation. Furthermore, the importance of MFN2 in cell differentiation is not confined to their role in mitochondrial dynamics and metabolism but also extends to other crucial functions, such as the regulation of ER [152]. This critical interplay, for example, is essential in the context of spermatogenesis since the disruption of these functions can compromise germ cell survival and differentiation, leading to male infertility [152].

Compared to MFN2, OPA1’s role is more confined to energy metabolism regulation, particularly in the context of mitochondrial OxPhos regulation. The structure of mitochondrial *cristae*, regulated by OPA1, plays a pivotal role in neuronal differentiation. *Cristae* support RCC organization, facilitating efficient electron transfer and ATP production. OPA1 remodeling is crucial for meeting the increased OxPhos demands of differentiating neurons, while OPA1 dysfunction impairs respiration and reduces differentiation efficiency, underscoring its significance in neuronal function and pathology [153,154].

Recent studies have emphasized that context-dependent changes in mitochondrial dynamics affect not only global processes like neuronal differentiation but also localized functions in specialized regions, such as dendrites, which are essential for synaptic modulation and plasticity [155,156,157]. For example, Kochan et al. showed that mitochondrial fusion promotes elongated mitochondria in dendrites of new neurons, and the absence of MFN1 or MFN2 disrupts synaptic plasticity in hippocampal neurons [155]. Additionally, the mitochondrial fusion/fission balance in dendrites of hippocampal neurons modulates synaptic activity by ensuring that energy supply meets synaptic demands. In high-metabolic regions, such as the apical dendrites, elongated, fused mitochondria are more prevalent, and their morphology is regulated by AMPK activation and phosphorylation of Mtfr1l, an OPA1-inhibiting protein [156]. Fusion-related mRNAs, such as those encoding MFN2 and OPA1, are selectively localized and stabilized in dendrites, axons, and optic nerves to meet local mitochondrial needs [40,156]. Factors like untranslated regions (UTRs), cis-regulatory elements, and RNA-binding proteins (RBPs) modulate the stabilization, transport, and translation of these mRNAs, ensuring mitochondrial proteins are produced at the right time and place [158,159]. For example, the presence of mitochondria-shaping mRNAs in synaptosomes and the subsequent translation of these proteins at synaptic sites suggest that local regulation at these regions is critical for their proper function [160]. Thus, the regulation of mitochondrial fusion and the localization of fusion-related mRNAs are dynamic, context-dependent processes essential for neuronal function, energy supply, and synaptic plasticity. Understanding these mechanisms could provide novel therapeutic targets for neurological disorders in which mitochondrial dysfunction and impaired synaptic plasticity play central roles [157].

## 7. Pathological Implications of MFN2 and OPA1 Mutations

Neurons are particularly vulnerable to mitochondrial dynamics defects due to their reliance on properly positioned mitochondria for ATP production and calcium buffering [9]. MFN2 is essential for mitochondrial transport along axons, while OPA1 is critical for respiratory efficiency and mitochondrial genome stability. Although both proteins regulate mitochondrial fusion, their mutations lead to distinct pathologies due to their unique roles and the specific vulnerabilities of neuronal subtypes [13].

### 7.1. MFN2 Mutations and Charcot-Marie-Tooth Disease Type 2A (CMT2A)

**Figure 2:**
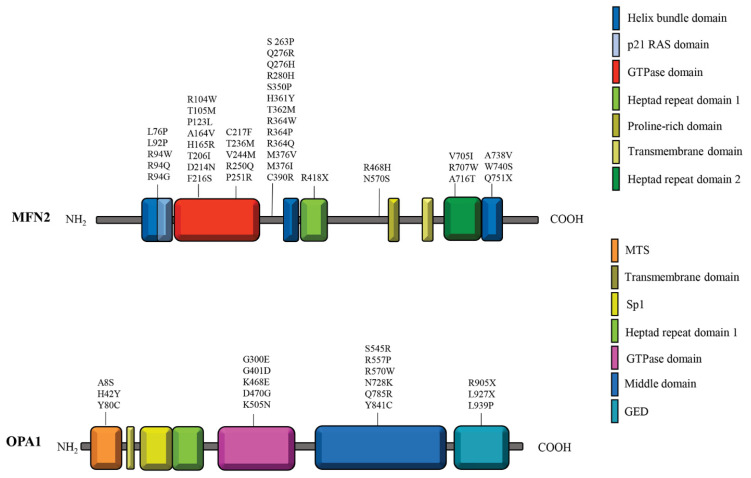
Secondary structure and commonly reported variants of MFN2 and OPA1 proteins. The schematic representation of MFN2 (above) highlights its structural domains: Helic bundle domain, p21 RAS domain, GTPase domain, heptad repeat domain 1 (HR1), a proline-rich domain, transmembrane domain, and heptad repeat domain 2 (HR2), each depicted in distinct colours. Below, OPA1 is illustrated with its key structural features: MTS (mitochondrial targeting sequence), a transmembrane domain, Sp1, heptad repeat domain 1, GTPase domain, middle domain, and GED (GTPase effector domain), also represented with corresponding colours. The most reported variants for both proteins are indicated in their respective domains.

MFN2 mutations are primarily associated with CMT2A, an autosomal dominant axonal neuropathy characterized by distal limb weakness, muscle atrophy, and sensory loss [161,162]. More than 60 pathogenic variants have been identified, often affecting the GTPase or coiled-coil domains (Figure 2). These mutations disrupt mitochondrial fusion, trafficking, and bioenergetics, leading to neuronal energy deficits and impaired calcium homeostasis [161,163]. Dysfunctional MFN2 impairs axonal transport, causing mitochondrial aggregation in the soma and reduced distribution to distal axons [164]. Both retrograde and anterograde transport are affected, leading to mis-localized mitochondria and compromised neuronal survival [123,165]. Loss-of-function MFN2 mutations result in fragmented mitochondrial networks with reduced OxPhos activity, while gain-of-function mutations may lead to abnormal mitochondrial morphology or toxic effects on neurons [166]. A dominant-negative mechanism often exacerbates dysfunction by interfering with wild-type MFN2 activity [167]. Additionally, mutant MFN2 proteins disrupt interactions with motor proteins, impairing mito-ER connectivity, calcium signaling, and lipid synthesis [168,169]. Interestingly, motor neurons expressing mutant MFN2 show apoptosis resistance due to p53 suppression, which enhances mitochondrial turnover as a compensatory mechanism [170,171] (Table 1). Potential therapeutic approaches for CMT2A focus on improving mitochondrial function and axonal transport. Strategies include modulating tubulin and Miro acetylation to facilitate mitochondrial motility and reducing stress-induced transcriptional changes affecting axonal organelle transport pathways [172].

### 7.2. OPA1 Mutations and Autosomal Dominant Optic Atrophy (ADOA)

Mutations in OPA1 are associated with autosomal dominant optic atrophy (ADOA), the most common mitochondrial optic neuropathy, characterized by retinal ganglion cell degeneration and progressive vision loss [173]. More than 370 pathogenic variants have been identified, including splicing mutations, frameshift deletions, and missense variants in the GTPase domain (Figure 2). These mutations disrupt inner membrane fusion and *cristae* organization, leading to respiratory dysfunction and mitochondrial fragmentation [174,175]. OPA1 haploinsufficiency reduces protein expression, sometimes triggering enhanced mitophagy and autophagy as compensatory responses to respiratory dysfunction [174,176,177,178,179,180,181,182]. Paradoxically, inhibiting autophagy in these cases has been shown to protect certain neuronal populations in mouse models [181,182]. However, no major alterations in the mitochondrial network were detected in other cases, correlating with low mitophagic/autophagic activity [175,183]. Some missense mutations operate through a dominant-negative mechanism, leading to excessive mitochondrial fragmentation despite normal OPA1 expression levels [175] (Table 2). Additionally, OPA1-deficient neurons exhibit decreased BNIP3 expression, a key regulator of mitophagy. Notably, restoring BNIP3 levels reactivates autophagic and mitophagic activity, highlighting its therapeutic potential [183,184]. Several studies have investigated therapeutic methods to restore mitochondrial fusion, regulate autophagy and mitophagy, and target compensatory mechanisms such as the downregulation of BNIP3. One potential strategy involves small molecules that enhance mitochondrial fusion and bioenergetics. For instance, Wang et al. (2012) identified M1, a compound that increased mitochondrial fusion and respiratory complex expression, offering a potential treatment avenue [185]. Additionally, Mdivi-1, a well-known DRP1 inhibitor, has been shown to increase OPA1 expression in cells carrying OPA1 variants, reducing excessive mitophagy and significantly improving cellular function [186]. Another approach focuses on direct OPA1 GTPase activation, which can promote mitochondrial fusion as a protective mechanism in various diseases [187]. Altogether, these therapeutic strategies hold promise for mitigating neurodegenerative disease progression and improving neuronal survival and function in disorders such as CMT2A and ADOA [10,170]. For further insights into therapeutic developments, readers are referred to recent comprehensive reviews [188,189,190].

**Table 1:** Mitochondrial alterations in CMT2A2 cellular models.

Cell Type	MFN2 Mutation	Remarks	References
Skin fibroblasts from patients with CMT2A	(1) P123L	(1) Mitochondrial fragmentation and abnormal mitochondrial accumulation around the nucleus	[163]
	(2) L92P	(2) Mitochondrial fragmentation	
Skin fibroblasts from 4 patients with CMT2A2	M21V	Reduced cellular respiration: uncoupling causing decreased ATP/OReduced ΔѰm	[191]
	R364Q		
	A166T		
Skin fibroblasts from patients with CMT2A	T105M	Mitochondrial morphology, mtDNA integrity and respiratory enzyme activities are unchangedExtensive mitochondrial fusion	[192]
	I213T		
	V273G		
Skin fibroblasts from patients with CMT2A	p.D210V	Respiratory chain defects	[193]
		Multiple mtDNA deletions	
		Defect in mtDNA damage repair system	
		Fragmentation of the mitochondrial network	
Skin fibroblasts derived from 4 patients with CMT2A	M376V	Mitochondrial respiratory chain dysfunctionDecreased mtDNA copy numberHigh levels of mtDNA depletion	[194]
	R707P		
	V226_S229del		
	Q74R		
Motor neurons derived from iPSCs of patients with CMT2A	R364W	Reduced mitochondrial trafficking with slower anterograde and retrograde velocities along axons	[195]
		Electrophysiological impairments including increased excitability, higher sodium current density, and reduced inactivation of voltage-dependent sodium and calcium channel	
Motor neurons derived from iPSCs of patients with CMT2A	A383V	Decreased respiratory chain activity: complexes II and III	[173]
		Reduced mitochondrial mass and mtDNA content	
		No mtDNA alterations	
		Decrease of mitochondrial trafficking leading to perinuclear aggregation	
		No survival and morphometric defects	
		Increased resistance to apoptosis	
		Increased autophagic and mitophagic flux	
Motor neurons derived from iPSCs of patients with CMT2A	R94Q	Abnormal mitochondrial morphology: shorter mitochondrial length within neurites, presence of abnormal mitochondria with loss of crista	[196]
		Reduction in the percentage of moving mitochondria	
		Decrease in ATP levels in neurites	
		Increased toxicity sensitivity to vincristine and paclitaxel	
Motor neurons derived from iPSCs of patients with CMT2A	R94Q	Hyper-connectivity: increase in burst rate	[172]
		Alterations in mitochondrial morphology: reduced mitochondrial elongation and increase in circularity	
		Impairment in axonal transport: decrease in the speed of mitochondria and lysosomes and the proportion of active mitochondria and lysosomes moving within the cells	
		Defects in OxPhos: decrease in mitochondrial basal respiration	
		Transcriptomic analysis: enrichment in PI3K-AKT signaling and respiratory chain pathway	
Skin fibroblasts derived from CMT2A patients	R364W	Mitochondrial mass and mtDNA levels are unchanged	[168]
	M376V	Moderate disturbances in Ca^2+^ homeostasis	
	W740S	Reduced ER-mitochondria contacts	
Skin fibroblasts from CMT2A patient	C217F	Mitochondrial mass and mtDNA levels and integrity unchanged	[197]
		Mitochondrial fragmentation	
		Reduced ΔѰm	
		Reduction of respiratory chain complexes activity	
		Transcriptomic analysis: enrichment in PI3K-AKT signaling	
		Reduced autophagy and increased cellular proliferation (mTORC2/AKT activation)	

**Table 2:** Mitochondrial alterations in ADOA/ADOA plus cellular models.

Cell Type	OPA1 Mutation	Remarks	References
Fibroblasts from 3 patients with ADOA plus	V903Gfs3,E221K QT86Sfs15, H957Y QT86Sfs*15, H957Y	Increased mitochondrial fragmentation	[178]
		Depletion of mtDNA	
		Altered mitochondrial localization	
		Increased mitophagy flux	
Fibroblasts from 7 ADOA patients	(1) S545R (2) R445H (3) I382M (4) R824* (truncated, low levels) (5) R557* (truncated, low levels)	(1), (2) Fragmented and punctiform mitochondria (3) Mild mitochondrial fragmentation (1), (2), (3) Loss of mitochondrial volume (1), (2) Mild uncoupling of oxidative phosphorylation (3), (4) Severe defects in oxidative phosphorylation Altered autophagy (4), (5) Mild mtDNA depletion	[175]
Lymphoblastoid cells derived from ADOA patients	P400A	Decreased mtDNA copy number	[198]
		Reduced levels of 4 mtDNA-encoded polypeptides	
		Respiratory capacity defects	
		ATP synthesis defects	
		Altered mitochondrial membrane potential	
		Increased ROS production	
		Increased apoptosis	
		Mitochondrial morphological defects (fragmentation and swelling)	
Lymphoblastoid cell lines from ADOA patient	c.1444–2A>C (splicing variants, deletion of the 15th exon in mRNA transcript, low protein levels)	Respiratory chain activity defects	[177]
		More punctate mitochondria clustered in the perinuclear region	
		No marked depletion of mtDNA or mitochondrial mass	
		Reduced ATP synthesis	
		Reduced ΔѰm	
		Increased ROS production	
		Increased mitophagy	
Skin fibroblasts from ADOA plus patient	H42Y	Mitochondrial mass unchanged	[199]
		mtDNA levels slightly increased	
		mtDNA integrity unchanged	
		Mitochondrial fragmentation	
		Reduced ΔѰm	
		Reduction of respiratory chain complexes activity	
		Increased ROS production	
		Transcriptomic analysis: enrichment in p21WAF1/CIP1 and p53 pathways along with downregulation of mitotic cell cycle genes	
		Reduced autophagy and increased expression of senescence markers (SA-β galactosidase; p53 and p21) associated with lower mTORC2 activity	

## 8. Distinct Cellular Outcomes of MFN2 and OPA1 Mutations

**Figure 3:**
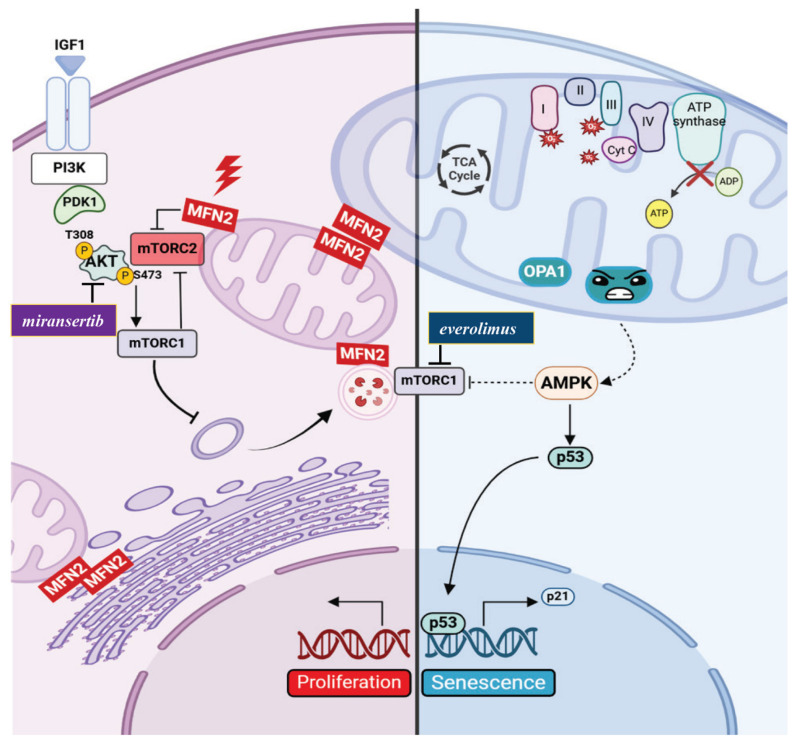
Schematic representation of altered processes in CMT2A and ADOA fibroblasts. Phosphatidylinositol 3-kinase (PtdIns3K) catalyzes the phosphorylation of phosphatidylinositol 4,5-bisphosphate (PtdIns2P) to phosphatidylinositol-3,4,5-trisphosphate (PtdIns3P), recruiting proteins such as 3-phosphoinositide-dependent kinase (PDK1). PDK1 phosphorylates AKT at Thr(308), while second phosphorylation at Ser(473) by mTORC2 fully activates AKT, promoting cellular proliferation. MFN2, localized at the outer mitochondrial membrane (OMM) or mitochondria-associated ER membranes (MAMs), suppresses mTORC2 kinase activity, thereby inhibiting AKT. Mutant MFN2 (red highlighting) loses this inhibitory function, leading to hyperactivated AKT, reduced autophagy, and increased proliferation. AKT promotes proliferation directly or via mTORC1 and inhibits autophagy through both mTORC1-dependent and independent mechanisms. Miransertib (shown in violet), an AKT inhibitor, restores autophagy and normalizes proliferation (left panel). AMPK, a serine-threonine kinase, acts as an energy sensor, maintaining cellular homeostasis by promoting survival and autophagy through mTORC1 suppression (right panel). OPA1, located in the inner mitochondrial membrane (IMM), regulates mitochondrial function. Mutant OPA1 (blue emoticon) induces mitochondrial depolarization (green mitochondrion), increases ROS production, and impairs the electron transfer chain (ETC). These dysfunctions lead to premature senescence and a decline in AMPK responsiveness (dotted line), reducing autophagy initiation and cell survival. OPA1-driven mitochondrial dysfunction contributes to p53 activation, which regulates stress response genes, including the cell cycle inhibitor p21. Increased p53 and p21 expression in OPA1-mutated cells promotes senescence. However, senescence and autophagy defects can be reversed by inhibiting mTORC1 using everolimus (shown in blue, right panel).

Recent studies (Zanfardino et al. 2022; 2024) have revealed distinct cellular effects of MFN2 and OPA1 mutations, particularly concerning mitochondrial dynamics, autophagy, and cellular phenotypes. Researchers analyzing primary fibroblasts carrying the MFN2^C217F^ and OPA1^H42Y^ mutations found significant mitochondrial fragmentation, depolarization, and impaired respiration, primarily due to decreased activity of respiratory chain complexes. These findings suggest a dominant-negative effect of the mutations, highlighting their detrimental impact on mitochondrial quality control [197,199,200,201]. Both MFN2 and OPA1 mutations reduced the formation of autophagosomes, indicating a shared defect in autophagy regulation. Interestingly, despite the accumulation of damaged and fragmented mitochondria, autophagy was not activated in either mutant fibroblast line. The mitophagy machinery remained intact, suggesting that the observed defects arise from impaired signaling or autophagosome biogenesis rather than a failure of the mitophagy process itself [197,199]. Transcriptomic profiling uncovered differential cellular responses between MFN2 and OPA1 mutations. In MFN2-mutated cells, genes associated with the PI3K/AKT signaling pathway and cell proliferation were upregulated, correlating with increased mTORC2/AKT activation, accelerated cell division, and reduced autophagy—consistent with the anabolic effects of PI3K/AKT/mTOR signaling [197,200,201] (Figure 3). These findings align with previous research demonstrating that MFN2 suppresses mTORC2 activity, influencing AKT phosphorylation at Ser473, a mechanism implicated in cancer growth and metastasis [202]. Additionally, in pancreatic cancer cells, MFN2 enhances autophagy by inhibiting PI3K/AKT/mTOR signaling, underscoring its role in maintaining MAM integrity and energy balance [203]. Conversely, OPA1-mutated fibroblasts exhibited a distinct profile, characterized by p21WAF1/CIP1 and p53 pathway activation and downregulation of mitotic cell cycle genes. This expression pattern suggests an early senescent phenotype, confirmed by elevated SA-β-Gal activity. Additionally, these cells showed increased ROS levels, indicating oxidative stress and mitochondrial dysfunction, which may drive senescence [199,204,205,206,207]. In OPA1 mutants, impaired mTORC2 activation, cellular senescence, and weakened autophagic responses were observed. Researchers suggested that mitochondrial dysfunction in OPA1-deficient cells contributes to premature ageing by suppressing AMPK responsiveness, a key regulator of autophagy and metabolic balance and preventing mTORC1 inhibition [199,208] (Figure 3). In support of this, previous studies in mice demonstrated that OPA1 deletion in muscle cells induces premature senescence and early death, further linking OPA1 mutations, mitochondrial dysfunction, and senescence [70,209]. Moreover, *OPA1* overexpression in Chang cells has been linked to mitochondrial fusion and senescence markers, reinforcing its role in ageing [210]. Similarly, aged mesenchymal stromal/stem cells (MSCs) and diseased postmitotic cells accumulate dysfunctional mitochondria with altered OPA1 levels, further emphasizing its role in aging and cellular decline [209,210,211,212].

### Therapeutic Implications

The PI3K/AKT/mTOR pathway has emerged as a key regulator of the divergent outcomes observed in MFN2 and OPA1 mutants. Pharmacological modulation of this pathway effectively normalized cellular phenotypes in mutant cell models. In MFN2-mutated cells, mTOR inhibition with torin1 restored normal cell proliferation, while AKT inhibition with miransertib improved both autophagy and cell proliferation [197,201]. In OPA1-mutated cells, mTORC1 inhibition with everolimus counteracted senescence and restored autophagy [199] (Figure 3). By inhibiting mTORC1, everolimus activates a cascade of downstream signaling events that promote autophagy, including the activation of key autophagy-related proteins and initiators, ultimately stimulating the formation of autophagosomes [199,213]. While the promotion of autophagy is linked to mTORC1 inhibition, the beneficial effects of everolimus on lifespan are attributed to both mTORC1 inhibition and the concurrent increase in mTORC2 activity, which regulates cell survival [214,215]. Furthermore, mTORC1 inhibition suppresses geroconversion, a process associated with cellular hypertrophy, lysosomal hyperactivation, and age-related dysfunction [216]. This suppression counteracts cellular aging but does not reverse cell cycle arrest caused by OPA1 mutations [199]. mTORC1 inhibition by everolimus reduces p21WAF1/CIP1 expression, indirectly increasing p53 levels and potentially inducing cell quiescence [217]. Quiescent cells, unlike senescent cells, can resume proliferation in response to appropriate signals [218,219,220]. These findings underscore the crucial link between mitochondrial dysfunction, organelle morphology, and cellular signaling. The PI3K/AKT/mTOR pathway is a key sensor of mitochondrial distress, orchestrating the subsequent cellular adaptations [172]. Targeting this pathway presents a promising therapeutic strategy to counteract the pathological consequences of MFN2 and OPA1 mutations and restore mitochondrial function [197,198,199].

## 9. Discussion

**Figure 4:**
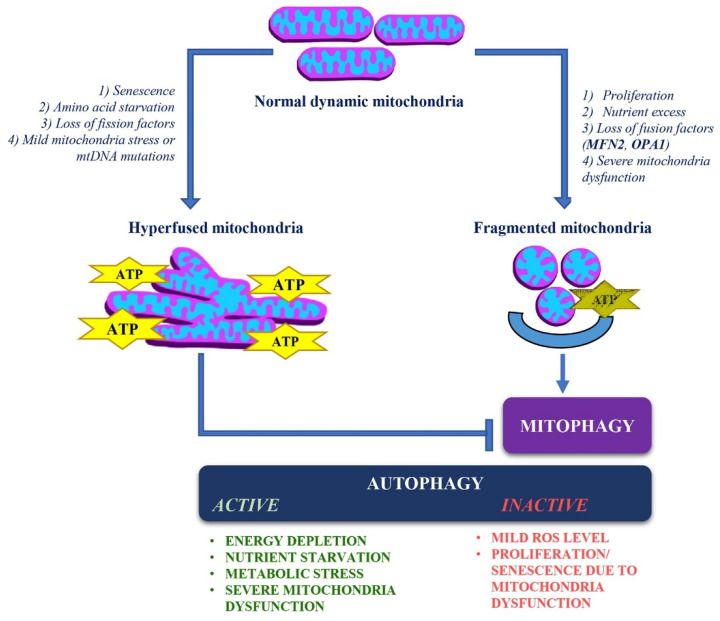
The balance between mitochondrial dynamics and cellular homeostasis. This illustration depicts the contrasting effects of different cellular conditions on mitochondrial dynamics and homeostasis. Mitochondrial hyperfusion can be triggered by cellular senescence, amino acid starvation, loss of fission factors, mild mitochondrial stress, or mitochondrial DNA (mtDNA) mutations. This state supports oxidative phosphorylation (OxPhos) and ATP production but simultaneously prevents the autophagic clearance of damaged mitochondria through mitophagy despite cellular stressors. Conversely, mitochondrial fragmentation occurs in response to cellular proliferation, nutrient excess, loss of fusion factors (such as MFN2 and OPA1), or severe mitochondrial dysfunction. This fragmentation promotes the selective removal of dysfunctional mitochondria via mitophagy. While mitophagy is typically a response to mitochondrial dysfunction, autophagy can also be activated by severe cellular stressors such as energy depletion, nutrient starvation, and metabolic stress. However, autophagy regulation is complex, and there are instances when, despite mitochondrial fragmentation, autophagy may not be activated, particularly in contexts such as cell proliferation (as seen in Charcot-Marie-Tooth disease type 2A) or premature senescence (observed in autosomal dominant optic atrophy). Another example is that under mild oxidative stress, mitophagy may still be induced, but non-selective autophagy does not necessarily occur in parallel.

MFN2 and OPA1 are crucial regulators of mitochondrial dynamics, playing key roles in balancing fusion and fission to maintain mitochondrial structure, quality control, and energy production in response to cellular conditions. MFN2 primarily functions at the OMM, while OPA1 operates at the IMM. Mitochondrial fusion plays a vital role in the exchange of mitochondrial components, which helps to prevent the buildup of defective proteins and mutated mtDNA and allows for functional complementation. During periods of metabolic stress, such as starvation or cellular senescence, mitochondrial fusion is enhanced and forms elongated networks. These networks support OxPhos and help cells evade mitophagy. In contrast, severe mitochondrial damage, excessive nutrient levels, or increased cell proliferation trigger mitochondrial fission, resulting in smaller mitochondria. This fission process facilitates the redistribution of mitochondria during mitosis and helps isolate damaged mitochondria for degradation through mitophagy (Figure 4). Although mitophagy is a specialized form of autophagy, it is independently regulated in response to mitochondrial damage. Despite this, both processes can interact to maintain cellular homeostasis. For instance, autophagy complements mitophagy by responding to broader metabolic cues, ensuring the health of the cell. The initiation of mitophagy is determined by the extent of mitochondrial damage or dysfunction and the associated energy status of the cell [221]. When mitochondria are not significantly harmed with respect to the high-energy costs, the cell tends to avoid their removal through mitophagy. The cell can recognize a decline in energy production resulting from mitochondrial changes. In response, it may initiate nonselective autophagy to acquire the nutrients needed for vital biosynthetic processes [222]. Research has indicated that mitochondrial dysfunction is associated with increased autophagic markers across different cellular disease models. For instance, in fibroblasts deficient in OxPhos, mitophagy is not increased, but there is an accumulation of autophagosomes, lysosomes and late autophagic vacuoles [223]. This accumulation is thought to result from a trigger of nonselective autophagy, which occurs in response to a pseudo-starvation state. When mitochondria are significantly impaired, cells can trigger mitophagy and activate a signaling pathway that encourages nonselective autophagy. Many studies highlight the role of specific mitophagy factors in regulating the overall autophagic response to mitochondrial damage [224]. Investigating how the absence of these factors affects autophagy initiation, rate, extent, and overall cell survival would provide valuable insights. Research conducted in yeast demonstrates that mitophagy is consistently accompanied by nonselective autophagy, indicating a strong connection between mitochondrial status and autophagic regulation [224]. However, it remains unclear whether mitochondrial fission is universally required for mitophagy or if it is only necessary under specific conditions. General autophagy-inducing situations, such as starvation, also trigger mitophagy, but the mechanisms governing selective mitochondrial degradation in these contexts remain uncertain. Furthermore, it is possible to identify conditions that allow mitophagy induction while non-selective autophagy is not triggered simultaneously. For example, in mammalian cells experiencing mild oxidative stress, DRP1-dependent mitophagy occurs without the activation of nonselective autophagy, suggesting that mitophagy can be regulated independently [225]. Additionally, it is important to note that mitochondrial fragmentation due to MFN2 and OPA1 mutations does not always lead to autophagy, as certain factors, such as cell proliferation (as seen in CMT2A) or premature senescence (as in ADOA), can suppress the autophagic process (Figure 4).

## 10. Conclusions

Mitochondrial dynamics, which involve a balance between fusion and fission, are vital for maintaining cellular homeostasis. MFN2 and OPA1 are key regulators of mitochondrial fusion; they not only influence mitochondrial shape but also play essential roles in bioenergetics, metabolic adaptation, and stress responses. Their regulation is particularly important during cellular reprogramming, where the transition between mitochondrial fragmentation and elongation determines different metabolic states. For instance, glycolysis is predominant in pluripotent cells, while OxPhos is favored during differentiation.

Disruptions in mitochondrial function, such as those seen with mutations in the *MFN2* and *OPA1* genes, contribute to neurodegenerative diseases like CMT2A and ADOA. Both conditions are characterized by mitochondrial fragmentation but lead to different cellular consequences. Dysfunction in MFN2 impairs mitochondrial transport and bioenergetics, resulting in reduced autophagy and increased cellular proliferation. This may promote the excessive survival of neuronal stem cells. In contrast, mutations in OPA1 hinder quality control mechanisms, resulting in cellular senescence and neurodegeneration. Despite their overlapping roles, MFN2 and OPA1 have distinct effects on cellular processes, particularly through their regulation of the mTOR/AKT pathway. This pathway mediates cellular responses to mitochondrial stress, energy status, and differentiation. These findings underscore the essential role of mitochondrial dynamics in cellular health and disease progression. Emerging therapeutic strategies aimed at restoring mitochondrial function—such as modulation of the mTOR pathway and enhancement of autophagy—offer promising approaches to address these diseases, with potential implications for other neurodegenerative conditions.

## 11. Future Directions

The divergent effects of MFN2 and OPA1 mutations raise critical questions about their shared and distinct roles in disease progression:

• Could the proliferative changes observed in CMT2A2 fibroblasts and cancer models of MFN2 dysfunction also occur in neuronal stem cells affected by CMT2A?
• Does senescence caused by OPA1 deficiency directly contribute to neurodegeneration in ADOA, and is this related to impaired autophagy?
• How does metabolic reprogramming influence the progression of mitochondrial diseases, and can interventions be implemented to restore the balance of autophagy and mitophagy?

To address these questions, future research should concentrate on advanced neuronal stem cell models, co-cultures, and 3D organoids that replicate in vivo environments. Investigating the interactions between mitochondrial dysfunction, metabolic shifts, and mTOR/AKT signaling within these systems will provide essential insights into disease mechanisms. Additionally, it will also offer the chance to model the disease in vitro, considering the specific patient genetic background. Pharmacological modulation of the mTOR/AKT pathway effectively normalized cellular phenotypes in mutant fibroblast models. Therefore, future studies should validate the therapeutic potential of these pharmacological strategies in additional in vitro or in vivo models. Since, to date, no cure/fully effective therapeutic strategy is available for these diseases, pharmacological therapy should be either supportive or curative [226,227]. Currently, pharmacological therapy has explored the use of similar pharmacological compounds, such as rapamycin, for its ability to induce mitochondrial biogenesis and enhance autophagy and mitophagy [228]. While beneficial in mitochondrial disease models, its use raises concerns about side effects, including immunosuppression and glucose intolerance [228,229]. However, preliminary evidence showed that everolimus improved immune response compared to rapamycin [229]. These findings support further studies in mice and humans to assess the long-term effects, safety, tolerability, and efficacy of rapamycin and rapalogs like everolimus. Limitations include variations in drug bioavailability, side effects, genetic background, blood–brain barrier permeability, and differing responses between tissues. The effectiveness of treatment may also vary based on factors like age, physical condition, and the tissues affected, with potential toxic side effects observed in mouse models [230].

The potential of mTOR inhibitors (such as everolimus and torin1) should be examined for their ability to restore autophagic flux, enhance mitochondrial quality control, and prevent neurodegeneration. The neuroprotective effects of autophagy-stimulating therapies, like torin1, have been observed in Parkinson’s models [231], though the impact on mitochondrial parameters has often been overlooked. Further research is needed to translate these therapies to the clinic, considering the specific characteristics of each mitochondrial disease.

## Data Availability

Not applicable.
